# Cavidad ósea de Stafne en una población venezolana. Estudio de prevalencia

**DOI:** 10.21142/2523-2754-1103-2023-162

**Published:** 2023-09-26

**Authors:** Sabrina Visicchio Cusati, Carlos Manresa Bruguera, Valeria Gallego Mengod, Mariana Villarroel-Dorrego

**Affiliations:** 1 Facultad de Odontología, Universidad Santa María. Caracas, Venezuela. sabrinavisicchio@gmail.com Universidad Santa María Facultad de Odontología Universidad Santa María Caracas Venezuela sabrinavisicchio@gmail.com; 2 Odontólogo, Esp. Cirugía Maxilofacial, MSc. Hospital General del Oeste Dr. José Gregorio Hernández. Caracas, Venezuela. manresa723@hotmail.com Hospital General del Oeste Dr. José Gregorio Hernández Caracas Venezuela manresa723@hotmail.com; 3 Odontólogo. Esp. Radiología Oral y Maxilofacial, Universidad Santa María. Caracas, Venezuela. imagen.valeriagallego@gmail.com Universidad Santa María Universidad Santa María Caracas Venezuela imagen.valeriagallego@gmail.com; 4 PhD Patología Bucal y Maxilofacial, Universidad Central de Venezuela. Caracas, Venezuela. reportesvillarroel@gmail.com Universidad Central de Venezuela Universidad Central de Venezuela Caracas Venezuela reportesvillarroel@gmail.com

**Keywords:** defecto óseo, fosa mandibular, panorámica, prevalencia, bone defect, mandibular fossa, panoramic, prevalence

## Abstract

**Objetivo:**

Determinar la prevalencia de la cavidad ósea de Stafne en una población venezolana y caracterizarla radiográficamente.

**Método:**

Estudio de tipo descriptivo y transversal. De 500 radiografías panorámicas, se seleccionaron aquellas que tenían imágenes radiolúcidas en mandíbula, circunscritas y no asociadas a ningún órgano dentario, así como aquellas que pudieran corresponderse con la fosa mandibular. Se cotejaron los resultados y se resolvieron las discrepancias por consenso. Las seleccionadas fueron caracterizadas radiográficamente. Las variables fueron comparadas usando la prueba t de Student para igualdad de medias, aplicando la prueba de Levene, y los valores de p < 0,05 fueron considerados estadísticamente significativos.

**Resultados:**

De las 500 radiografías evaluadas, 22 presentaron imágenes correspondientes a cavidades óseas de Stafne, lo que representó el 4,4%. De estas, 5 se presentaron de forma bilateral (22,7%) y 17 (77,3%) de forma unilocular. Entre las características radiográficas estudiadas, se presentaron con mayor prevalencia imágenes ubicadas en la zona posterior (81,81%), sin esclerosis (54,54%), continuas a la basal mandibular, con forma redonda (59,09%) y de radiolucidez parcial (72%). estas fueron más comunes en pacientes masculinos, con un 63,63% del total.

**Conclusiones:**

Nuestros resultados muestran una prevalencia mayor de la cavidad ósea de Stafne en una población venezolana que la reportada en estudios aplicados a otras poblaciones.

## Abstract (en)

**Objective:**

To determine the prevalence of the Stafne´s bone cavity in a Venezuelan population and to characterize it radiographically.

**Method:**

Descriptive and cross-sectional study. Of 500 panoramic radiographs, those that had radiolucent images in the mandible, circumscribed and not associated with any dental organ, as well as those that could correspond to the mandibular fossa, were selected. Results were collated and discrepancies resolved by consensus. The selected ones were characterized radiographically. The variables were compared using the student's t test for equality of means, applying the Levene test, values of p <0.05 were considered statistically significant.

**Results:**

Of the 500 radiographs evaluated, 22 presented images corresponding to deep mandibular fossa or Stafne bone cavities, which represented 4.4%. Of which 5 presented bilaterally (22.7%) and 17 (77, 3%) unilocularly. Among the radiographic characteristics studied, images located in the posterior area (81.81%), without sclerosis (54.54%), continuous to the basal mandibular area, round shape (59.09%) and partial radiolucency (72%) were presented with a higher prevalence). They were more common in male patients with 63.63%.

**Conclusions:**

Our results show a higher prevalence of Stafne's bone cavity in a Venezuelan population than that reported in other studies in other populations.

## INTRODUCCIÓN

La cavidad ósea de Stafne, también llamada defecto o quiste óseo de Stafne, se presenta como una variante anatómica poco frecuente, radiolúcida, bien delimitada, que usualmente se presenta en la región posterior mandibular, entre el canal mandibular y el borde inferior de la mandíbula. Fue descrita por primera vez en 1942, por Edward C. Stafne, quien, durante su trabajo en la Clínica Mayo, reportó 35 casos asintomáticos en los que describió la radiolucidez de las imágenes ubicadas debajo del canal mandibular, en la región posterior del cuerpo, y las asoció con la invaginación lingual del hueso cortical ^1^^-^^4^.

La etiología de esta variante es incierta; sin embargo, se han planteado como teorías un defecto congénito, la hipertrofia de una glándula salival, la erosión generada por compresión vascular o, incluso, la calcificación incompleta del cartílago de Meckel durante la osificación de la mandíbula ^5^^-^^13^. La teoría de la reabsorción por presión es una de las más aceptadas y explica que esta ocurre por la presión de la glándula salival sobre la superficie lingual de la mandíbula. Stafne afirmó que estas concavidades fueron causadas por la hipoplasia del hueso mandibular, durante su etapa de crecimiento y desarrollo. La isquemia ósea y la presión vascular anormal de la arteria facial son algunas de las otras hipótesis descritas ^4^.

La glándula submandibular se encuentra relacionada directamente con la superficie media del cuerpo de la mandíbula y, generalmente, causa una depresión en el hueso denominada fosa submandibular. En algunos casos, la fosa submandibular puede ser más profunda de lo habitual y se invagina hacia el espacio medular de la mandíbula, lo que genera el defecto óseo de Stafne ^14^. La glándula submandibular se relaciona mayormente con la variante posterior de estas imágenes, mientras que la glándula sublingual lo hace con la variante anterior y la glándula parótida, con las dos variantes de la rama ascendente de la mandíbula ^15^. Tanto las fosas mandibulares profundas como la cavidad ósea de Stafne producen imágenes de similares características radiográficas cuando son observadas en radiografías bidimensionales. 

El propósito de este estudio fue determinar la prevalencia de imágenes que se correspondan con cavidades óseas de Stafne en una población venezolana y caracterizarla radiográficamente en ortopantomografías.

## MATERIALES Y MÉTODO

El estudio fue aprobado por el comité de ética local. Se realizó un estudio de paradigma cuantitativo, de tipo descriptivo y transversal. Se seleccionaron 500 radiografías panorámicas de pacientes venezolanos provenientes de un mismo centro radiológico tomadas en un período de 24 meses. 

Posteriormente, 2 evaluadores, previamente calibrados, seleccionaron aquellas radiografías que tenían imágenes radiolúcidas en mandíbula circunscritas y no asociadas a ningún órgano dentario. Luego, se cotejaron los resultados y se resolvieron las discrepancias por consenso. 

Las radiografías seleccionadas fueron evaluadas posteriormente a juicio de experto y las seleccionadas, finalmente, fueron clasificadas según su ubicación y las características radiográficas descritas en el estudio de Hisatomi ^12^. Datos como la edad y el sexo del paciente fueron incluidos. La identidad de los pacientes fue resguardada en todo momento.

Las variables fueron comparadas usando la prueba t de Student para igualdad de medias, aplicando la prueba de Levene y los valores de p < 0,05 fueron considerados estadísticamente significativos.

## RESULTADOS

**Figura 1:**
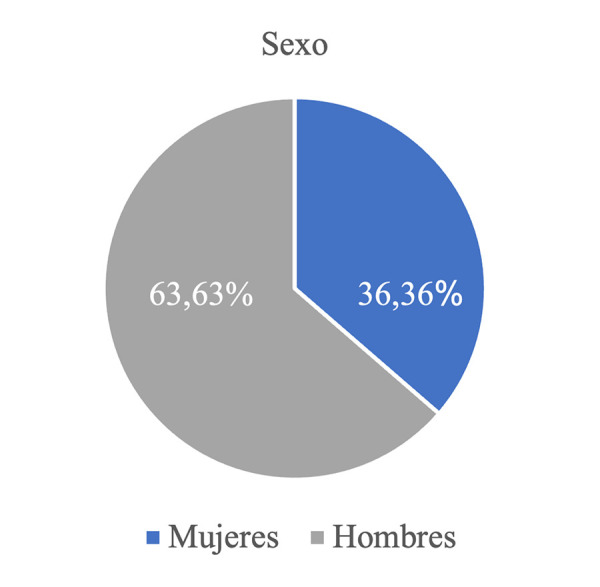
Prevalencia de cavidades óseas en la muestra, según sexo

De las 500 radiografías evaluadas, 22 presentaron imágenes correspondientes a cavidades óseas de Stafne, lo que representó el 4,4%. Se presentaron 5/22 de forma bilateral (22,7%) y la prevalencia fue mayor en los pacientes masculinos, con un 63,63% (Fig. 1). 

**Figura 2:**
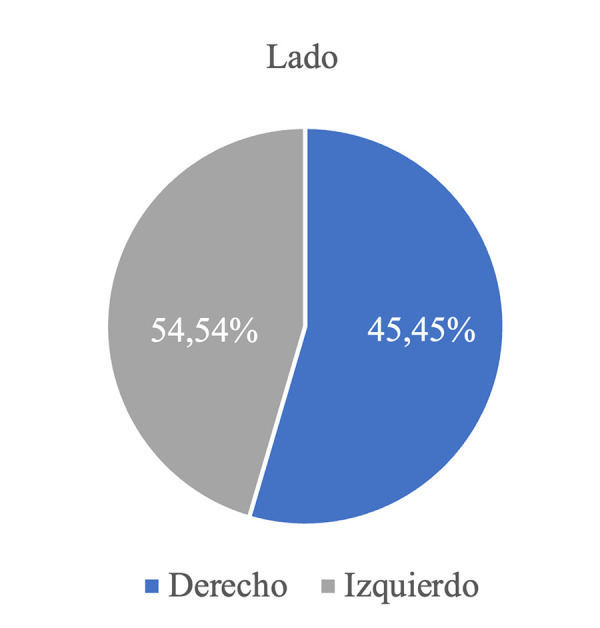
Prevalencia de cavidades óseas en la muestra, según lado

**Figura 3:**
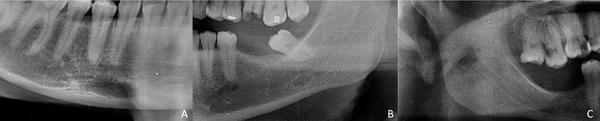
Clasificación de acuerdo con la ubicación: A. Variante anterior; B: Variante posterior; C. Variante de la rama mandibular

En el lado derecho, se presentó en un 54,54%, y el lado izquierdo, en un 45,45% (Fig. 2). La media de la edad fue de 33,5 +/- 6,9 años. Con respecto a su ubicación, las variantes más comunes fueron la posterior (81,81%), anterior (13,63%) y en la rama mandibular (4,54%) (Fig. 3). 

**Figura 4:**
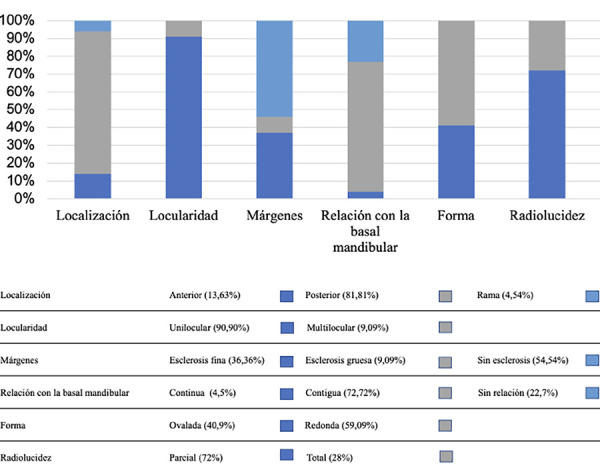
Características radiográficas encontradas en la muestra, según Hisatomi ^12^

Se encontró contigua a la basal mandibular en un 72,72%, continua en un 4,5% y sin relación en un 22,7%. En cuanto a la forma, las imágenes fueron redondas y ovaladas en un 59,09% y un 40,9%, respectivamente. La presentación unilocular fue la más común, con un 90,90%, a diferencia de la multilocular, que solo estuvo presente en un 9,09%. Con respecto a sus márgenes, se presentaron sin esclerosis en el 54,54% de los casos, con esclerosis fina en el 36,36% y con eslerosis gruesa en el 9,09% (Fig. 4). Asimismo, se presentó con radiolucidez total en un 28% y parcial en un 72%.

De manera interesante, en el sexo masculino se presentó exclusivamente en la ubicación posterior, mientras que el femenino se distribuyó entre anterior y posterior (p = 0,0001), a diferencia de la locularidad, donde en el sexo masculino se presentó mayormente de forma multilocular y en el femenino, exclusivamente unilocular (p = 0,016). También se observó que la ubicación en el lado derecho se presentó con mayor prevalencia en el sexo femenino (p = 0,022). Con respecto a los márgenes, se pudo apreciar un predominio en el lado izquierdo, donde se presentaron mayormente con esclerosis gruesa y sin esclerosis (p = 0,015). Con respecto a la forma, todos los casos multiloculares se presentaron ovalados y todas las presentaciones de forma redonda fueron uniloculares (p = 0,003).

Al relacionar la forma con la radiolucidez, las imágenes se presentaron de forma redonda con mayor prevalencia cuando tenían radiolucidez parcial (p = 0,030). Con respecto a la locularidad, los casos multiloculares se presentaron exclusivamente en pacientes de sexo masculino (p = 0,0001), y en las presentaciones uniloculares fue totalmente radiolúcidos en todos los casos (p = 0,035). La radiolucidez parcial se presentó con mayor predominio en el lado izquierdo (p = 0,030), mientras que en el lado derecho se presentó más comúnmente la forma ovalada.

## DISCUSIÓN

En nuestro estudio, la prevalencia de la cavidad ósea de Stafne (4,4%) fue mayor que la reportada en diferentes investigaciones. La descrita por Voss ^13^ oscila entre el 0,1% y el 1,3%, al igual que en los estudios realizados por Arya ^16^, Cavalgante ^17^ y Páucar ^18^, en lo que se presentó en un 0,03%, 0,08% y 0,13%, respectivamente. A pesar de que en el metaanálisis de Chaweeborisuit ^1^ se afirma que la incidencia es mayor en estudios basados en tomografía y excavaciones arqueológicas, otros autores señalan una mayor prevalencia cuando las imágenes son evaluadas en radiografías panorámicas en comparación con las encontradas en tomografías ^17^^-^^21^. Los criterios para discernir entre una fosa mandibular profunda de una verdadera cavidad de Stafne en una imagen bidimensional podría influir también en este porcentaje, razón por la cual incluimos ambas entidades en nuestro estudio; esto podría explicar la mayor prevalencia reportada.

En el trabajo realizado por Hisatomi ^12^, en el que se evaluaron 91 casos de cavidades óseas de Stafne, predominó la presencia de esta en el sexo masculino, al igual que nuestro estudio y en todos los consultados ^1^^,^^2^^,^^12^^,^^16^^-^^19^^,^^21^. Curiosamente, en el metaanálisis de Chaweeborisuit ^1^, los valores fueron mayores en individuos pertenecientes a Sudamérica, como la población estudiada en nuestro estudio, seguidos por europeos, norteamericanos y asiáticos.

En cuanto a la ubicación y las características radiográficas más comunes encontradas en nuestro estudio, los resultados coinciden con la zona del cuerpo mandibular y la presentación única, al igual que lo reportado por Arya y Páucar ^16^^,^^18^.

En relación con la edad, en nuestro estudio se presentó en una población más joven, lo que contrasta con lo descrito en la literatura revisada, donde es más comúnmente encontrada entre la quinta y sexta décadas de vida, mientras que en la nuestra se presentó con mayor frecuencia en la tercera década ^12^^,^^16^^,^^19^. Sin embargo, debido a la etiología de estos defectos o las variaciones anatómicas que pueden presentarse, consideramos que las diferencias en la edad pueden deberse más a un factor sociodemográfico de la población estudiada que a una relación entre la imagen y la edad *per se*.

En nuestro estudio, los márgenes de las imágenes se presentaron mayormente sin esclerosis, a diferencia de las características radiográficas presentadas por Hisatomi, donde prevaleció la esclerosis gruesa. Para ellos, la presentación sin esclerosis podría asociarse con la ubicación contigua con el borde mandibular. Sin embargo, los resultados coinciden en que la radiolucidez parcial tuvo mayor prevalencia, ya que en las radiografías puede estar relacionada con la preservación ósea de la pared vestibular ^12^.

La importancia de la correcta identificación de estas imágenes radica en su parecido con ciertos quistes y tumores de la mandíbula, entre ellos los quistes residual, radicular, queratoquiste odontogénico, dentígero y ameloblastoma, como principales diagnósticos diferenciales. Radiográficamente, estas lesiones se presentan como imágenes radiolúcidas bien delimitadas, redondas u ovaladas en su forma, que pueden asemejarse a la cavidad ósea de Stafne. El quiste residual, además de encontrarse más comúnmente en el maxilar, suele ubicarse en una zona asociada con un diente extraído, mientras que el quiste dentígero debe estar asociado con la corona de un diente impactado ^22^. El quiste periapical, por su parte, se desarrolla en el periápice de un diente no vital, por lo que estas dos características son mandatorias para su diagnóstico. 

Con respecto al queratoquiste odontogénico, radiográficamente, también se observa como una imagen radiolúcida bien circunscrita que puede presentarse uni o multilocular, con bordes radiopacos delgados, a diferencia del defecto óseo que en la mayoría de los casos se presenta sin esclerosis y siempre unilocular ^23^. Es necesario también hacer el diagnóstico diferencial con respecto al ameloblastoma, que se presenta más comúnmente de forma multilocular, con una apariencia de pompas de jabón y que causa reabsorción de las piezas dentarias relacionadas y expansión de la cortical ósea, pero que en estadios iniciales pudiera presentarse con características similares al defecto ^24^.

Finalmente, las fosas mandibulares profundas pueden producir imágenes en radiografías panorámicas similares a las cavidades óseas de Stafne, lo cual aumenta la probabilidad de ser encontradas durante el examen radiográfico de rutina. A pesar de estar descritas desde hace más de 80 años, estas cavidades siguen siendo desconocidas por muchos odontólogos.

Su baja frecuencia, sumada al desconocimiento sobre ellas, hace que pasen desapercibidas o, en muchos casos, sean consideradas desde el inicio como una entidad patológica, lo que puede generar ansiedad en el paciente hasta que es diagnosticado correctamente o, eventualmente, ser sometido a cirugías innecesarias para la toma de biopsias.

Los hallazgos en radiografías panorámicas de estas imágenes deben ser cuidadosamente evaluados considerando todas sus características. Estudios de imagen como la tomografía o la resonancia magnética son sumamente útiles en muchos casos para confirmar estas sospechas. Conocer sus características más frecuentes nos permitirá llegar más fácilmente al diagnóstico correcto.

## CONCLUSIONES

La prevalencia de la cavidad ósea de Stafne en una población venezolana fue mayor que la reportada en otros estudios en otras poblaciones. Por ello, se recomienda insistir a nivel educativo en la consideración de la cavidad de Stafne como una imagen no patológica y poco frecuente observada en las radiografías panorámicas, y correlacionar a nivel asistencial estos hallazgos radiográficos con la clínica, la epidemiología y estudios de imagen que permitan descartar la cavidad como diagnóstico presuntivo antes de invadir al paciente.

## Fuente de financiamiento:

El trabajo fue totalmente autofinanciado.
